# Pharmacological Targeting of STING-Dependent IL-6 Production in Cancer Cells

**DOI:** 10.3389/fcell.2021.709618

**Published:** 2022-01-11

**Authors:** Sumaiah S. Al-Asmari, Aleksandra Rajapakse, Tomalika R. Ullah, Geneviève Pépin, Laura V. Croft, Michael P. Gantier

**Affiliations:** ^1^ Centre for Innate Immunity and Infectious Diseases, Hudson Institute of Medical Research, Clayton, VIC, Australia; ^2^ Department of Molecular and Translational Science, Monash University, Clayton, VIC, Australia; ^3^ School of Biomedical Sciences, Centre for Genomics and Personalised Health, Cancer and Ageing Research Program at the Translational Research Institute, Queensland University of Technology (QUT), Brisbane, QLD, Australia

**Keywords:** STING, IL-6, cancer, DNA damage, STING inhibitor, ERK1/2, Non-canonical STING

## Abstract

Activation of the STING pathway upon genotoxic treatment of cancer cells has been shown to lead to anti-tumoral effects, mediated through the acute production of interferon (IFN)-β. Conversely, the pathway also correlates with the expression of NF-κB-driven pro-tumorigenic genes, but these associations are only poorly defined in the context of genotoxic treatment, and are thought to correlate with a chronic engagement of the pathway. We demonstrate here that half of the STING-expressing cancer cells from the NCI60 panel rapidly increased expression of pro-tumorigenic IL-6 upon genotoxic DNA damage, often independent of type-I IFN responses. While preferentially dependent on canonical STING, we demonstrate that genotoxic DNA damage induced by camptothecin (CPT) also drove IL-6 production through non-canonical STING signaling in selected cancer cells. Consequently, pharmacological inhibition of canonical STING failed to broadly inhibit IL-6 production induced by CPT, although this could be achieved through downstream ERK1/2 inhibition. Finally, prolonged inhibition of canonical STING signaling was associated with increased colony formation of MG-63 cells, highlighting the duality of STING signaling in also restraining the growth of selected cancer cells. Collectively, our findings demonstrate that genotoxic-induced DNA damage frequently leads to the rapid production of pro-tumorigenic IL-6 in cancer cells, independent of an IFN signature, through canonical and non-canonical STING activation; this underlines the complexity of STING engagement in human cancer cells, with frequent acute pro-tumorigenic activities induced by DNA damage. We propose that inhibition of ERK1/2 may help curb such pro-tumorigenic responses to DNA-damage, while preserving the anti-proliferative effects of the STING-interferon axis.

## Introduction

Upon activation by cytoplasmic DNA, cyclic guanosine monophosphate–adenosine monophosphate (cGAMP) synthase (cGAS) synthesizes cGAMP, which binds to the adaptor protein STING (stimulator of interferon [IFN] genes) (Zhang et al., 2013). This results in STING translocation from the ER to the Golgi, where it is palmitoylated to recruit TANK-binding kinase 1 (TBK1) and the inhibitor of nuclear factor kappa-B kinase subunit epsilon (IKKε) (Mukai et al., 2016; Balka et al., 2020). This in turn activates IRF3 and NF-κB transcriptional programs, culminating in the production of IFN-β and pro-inflammatory cytokines such as IL-6 and TNFα, respectively.

In addition to its immune function in the sensing of cytosolic pathogenic DNA, cGAS can initiate immune responses to endogenous nuclear and mitochondrial DNA (Dou et al., 2017; Wu et al., 2019). Such cGAS sensing of cytosolic DNA arising from genome instability promotes senescence and replicative crisis, aimed at eliminating pre-cancerous cells (Dou et al., 2017; Glück et al., 2017; Nassour et al., 2019). Accordingly, since cancer cells have deregulated cell cycle checkpoints they frequently harbor cytoplasmic DNA, which is increased further upon genotoxic damage and radiotherapy exposure, and can lead to cGAS-STING activation (Chen et al., 2017; Dou et al., 2017; Harding et al., 2017; Mackenzie et al., 2017; Bakhoum et al., 2018; Nassour et al., 2019; Carozza et al., 2020); (Marcus et al., 2018; Schadt et al., 2019; Carozza et al., 2020).

While DNA damage-driven GAS-STING cell-intrinsic engagement in cancer cells has been shown to be involved in the recruitment of immune cells to promote anti-cancer activities, through the engagement of the IRF3/IFN-β arm (Ho et al., 2016; Takashima et al., 2016; Harding et al., 2017; Vanpouille-Box et al., 2017; Yamazaki et al., 2020; Suter et al., 2021; Tian et al., 2021), there is also evidence that chronic activation of the pathway can drive tumorigenesis and metastasis (Ahn et al., 2014; Lemos et al., 2016; Bakhoum et al., 2018). The latter is aligned with a correlation between cGAS-STING expression in human cancers and pro-inflammatory NF-κB signatures, including the expression of IL-6 (Dou et al., 2017; Bakhoum et al., 2018). Such NF-κB signals can fuel the resistance to the DNA damage (Didonato et al., 2012), and directly contribute to the growth of cancer cells (Chen et al., 2016; Bakhoum et al., 2018). As such, IL-6 production results in autocrine and paracrine activation of STAT3 signaling that promotes survival of cancer cells in response to DNA damage and pro-apoptotic mediators such as TNFα (Li et al., 2012; Yun et al., 2012). Further, IL-6 directly inhibits the IRF3/IFN-β arm of STING signaling in selected cancer cells, alleviating the tumor suppressive effects of the pathway *in vivo* (Wu et al., 2017; Suter et al., 2021).

Albeit currently proposed to be associated with chronic STING activation (Decout et al., 2021), little is known of the mechanisms regulating the engagement of STING-dependent pro-inflammatory NF-κB factors in the context of acute genotoxic treatment of cancer cells. A recent study reported the existence of a non-canonical STING pathway, rapidly driving IL-6 production with minimal IFN-β production upon DNA damage resulting from topoisomerase-2 inhibition in HaCaT keratinocytes (Dunphy et al., 2018). This non-canonical STING pathway was independent of cGAS/cGAMP/TBK1 and did not require translocation from the ER to the Golgi (Dunphy et al., 2018). However, whether this non-canonical STING pathway is involved in the response to acute genotoxic treatment of cancer cells is currently unknown.

Following on the observation that pharmacological inhibition of STING reduced IL-6 production upon topoisomerase 1 inhibition in mouse TC-1 cancer cells, we decided to broadly interrogate the role of STING signaling in the IL-6 response to acute DNA damage in human cancer cells. Our results collectively support a direct role for STING signaling in the frequent IL-6 production in response to genotoxic treatment of cancer cells, most often independent of a marked IRF3 signature. As such, we demonstrate that both canonical and non-canonical STING signaling can participate in the rapid IL-6 production seen upon DNA damage in different cancer cells, indicating that the pro-tumorigenic activities of the pathway are not limited to its chronic engagement. We also provide evidence that ERK1/2 pharmacological inhibition may provide therapeutic opportunities to limit production of IL-6 upon genotoxic treatment, while preserving the anti-proliferative effects of the STING-interferon axis.

## Materials and Methods

### Cell Culture and Treatments

Human osteosarcoma MG-63 and HOS cells were purchased from ATCC (#CRL-1427 and #CRL-1543, respectively) and grown in ATCC-formulated Eagle’s Minimum Essential Medium, supplemented with 10% heat-inactivated fetal bovine serum (Thermo Fisher Scientific) and 1 × antibiotic/antimycotic (Thermo Fisher Scientific). PC-3 cells purchased from ATCC (#CRL-1435) and BT-549 breast ductal carcinoma cells (a kind gift from Prof S. Lakhani) were grown in Roswell Park Memorial Institute (RPMI) 1,640 plus L-glutamine medium (Life Technologies) complemented with 1x antibiotic/antimycotic and 10% heat inactivated fetal bovine serum (referred to as complete RPMI). TC-1 cells (kind gift from Prof. N. McMillan) and HaCaT cells (wild type–kind gift from Prof. S.M. Jane) were cultured in Dulbecco’s modified Eagle’s medium plus L-glutamine supplemented with 1 × antibiotic/antimycotic (Thermo Fisher Scientific) and 10% heat-inactivated fetal bovine serum (referred to as complete DMEM). SK-OV-3 ovarian carcinoma cells (a kind gift from Prof J. Hooper) were cultured in McCoy’s medium (Thermo Fisher Scientific) plus L-glutamine and 10% heat inactivated fetal bovine serum. MDA-MD-231 and HS-578T breast carcinoma cells (a kind gift from Prof S. Lakhani) were cultured in complete DMEM. HaCaT, MDA-MD-231, SK-OV-3 and BT-549 were authenticated using the *GenePrint®* 10 System kit from Promega. All the cells were cultured at 37°C with 5% CO_2_. Cell lines were passaged 2–3 times a week and tested for *mycoplasma* contamination on a routine basis by PCR. For clonogenic assays, ∼1,500 cells were added per well of a 6-well plate, and the drugs/medium changed every 2–3 days. After the indicated times, cells were fixed with 10% formalin and stained with 0.1% crystal violet (w/v) in 20% ethanol, before several thorough H_2_O washes.

Further methods are available in Supplementary Materials and Methods.

## Results

### Pharmacological Inhibition of Canonical STING Signaling Decreases CPT-Induced IL-6 in Mouse TC-1 Cells

**FIGURE 1:**
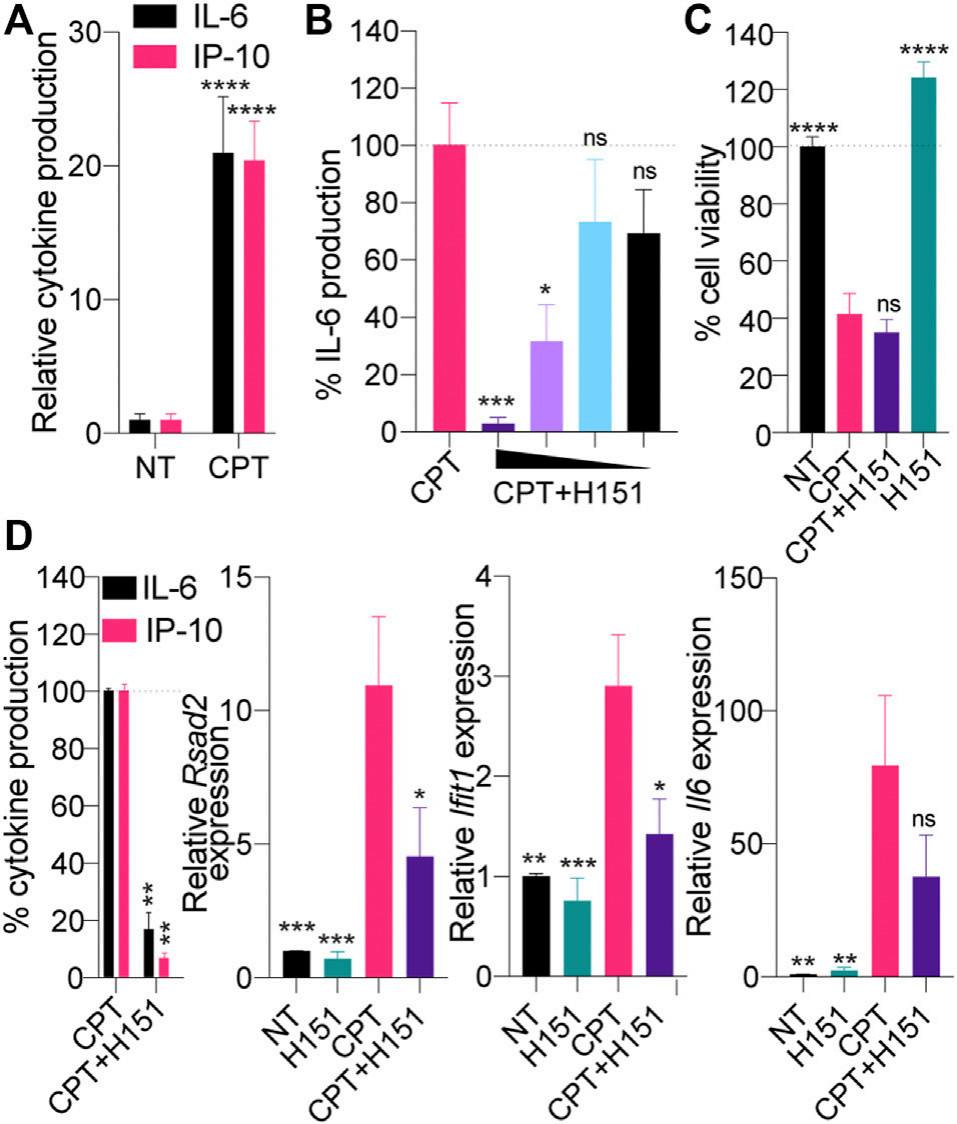
**(A)** TC-1 cells were treated with 0.5 μM CPT for 48 h, and IL-6 and IP-10 levels in supernatants were determined by ELISA. Cytokine levels were normalized to the non-treated (“NT”) condition after background correction with the NT condition. Data shown are averaged from three independent experiments in biological replicate (±s.e.m. and ordinary two-way ANOVA with Sidak’s multiple comparison tests). **(B, C)** TC-1 cells were treated with 0.5 μM CPT in the presence of decreasing amount of H151 [7.2, 3.6, 1.8 or 0.9 μM for **(B)**, 3.6 μM for **(C)**] for 48 h and IL-6 levels in supernatants were determined by ELISA **(B)** or cell viability assessed with resazurin assay **(C)**. B) IL-6 levels were normalized to the “CPT only” and are shown as percentages. Data shown are averaged from two independent experiments in biological triplicate (±s.e.m. and ordinary one-way ANOVA with Dunnett’s multiple comparison tests to the “CPT only” condition). **(C)** Data were normalized to the NT condition, after background correction with blank condition. Data shown are averaged from three independent experiments in biological replicate (±s.e.m. and ordinary one-way ANOVA with Dunnett’s multiple comparison tests to the “CPT only” condition). **(D)** TC-1 cells were treated with 0.5 μM CPT with or without 3.6 μM H151 for 48 h, and IL-6 and IP-10 levels in supernatants were determined by ELISA, while cell lysates were processed for RNA purification. Left: cytokine levels were normalized to the “CPT only” condition and are shown as percentages. Right: Expression of the panel of 3 mouse IFN-driven genes was analyzed by RT-qPCR. Expression of the indicated genes was reported relative to 18 s expression and divided further by the mean of the NT condition. Data shown are averaged from three independent experiments in biological replicate. Left: ± s.e.m. and Mann-Whitney U tests are shown; right: ± s.e.m. and ordinary one-way ANOVA with Dunnett’s multiple comparison tests to the “CPT only” condition. *p≤0.05, **p≤0.01, ***p≤0.001, ****p≤0.0001 and “ns” is non-significant.

We have recently reported that expression of the simian virus 40 (SV40) large T antigen could lead to potentiation of cGAS-STING engagement in cells treated with low-dose topoisomerase 1 inhibition with camptothecin (CPT) treatment (Pépin et al., 2017a). To broaden our observations to other viral oncogenes, we initially investigated whether CPT could induce STING-dependent signaling in mouse epithelial TC-1 cancer cells, which were co-transformed with HPV-16 E6 and E7 and c-Ha-ras oncogenes (Lin et al., 1996). Focusing on IL-6 and IP-10 production as surrogate markers of the NF-κB and IRF3 branches of STING activation, respectively (Pépin et al., 2017b; Dunphy et al., 2018), we first showed that low-dose CPT significantly induced the production of both cytokines in TC-1 cells (Figure 1A).

To implicate STING directly in this response to CPT, we repeated the experiments above using a recently reported pharmacological inhibitor of canonical STING, by preventing its palmitoylation, referred to as H151 (Haag et al., 2018). CPT-driven IL-6 production by TC-1 cells was significantly inhibited by H151 in a dose-dependent manner, without increasing further the cell death induced by CPT (Figures 1B,C). Accordingly, while H151 decreased production of IP-10 and IL-6 protein by ELISA, we also observed a decrease in expression of interferon-stimulated genes (ISGs) *Rsad2* and *Ifit1,* along with *Il-6* at the mRNA level by RT-qPCR (Figure 1D).

### Divergent Induction of IL-6 and ISGs in Response to DNA Damage in Human Cancer Cells

**FIGURE 2:**
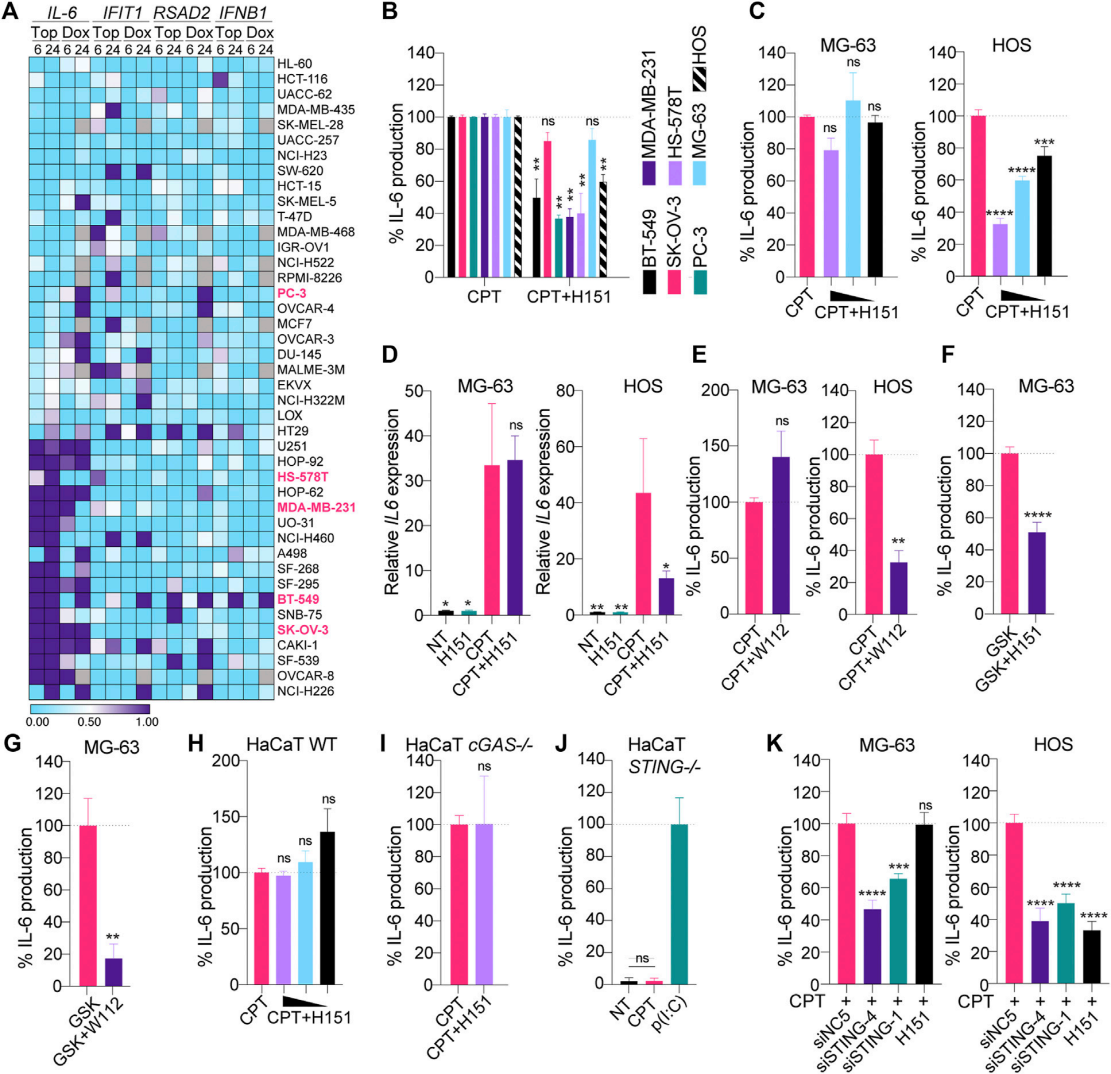
**(A)** Selected NCI-60 cell lines expressing STING (see Supplementary Table S1) were treated for 6 or 24 h with 1 μM topotecan (Top) or doxorubicin (Dox) and analyzed by microarray as reported in the NCI Transcriptional Pharmacodynamics Workbench (Monks et al., 2018). The heatmap shows the log_2_ fold change to NT condition (the values below 0.5 are blue and the values above are purple). Missing values are shown in grey. **(B)** Indicated cell lines were treated with CPT (see Materials and Methods for dosage used) with or without 3.6 μM H151 for 24 (BT-549, HS-578T, MDA-MB-231, PC-3 and SK-OV-3 cells) or 48 h (MG-63 and HOS cells), and IL-6 levels in supernatants were determined by ELISA. IL-6 levels were normalized to the “CPT only” condition and are shown as percentages. Data shown are averaged from three independent experiments in biological replicate (±s.e.m. and Mann-Whitney U tests are shown). **(C)** MG-63 and HOS were treated with CPT with or without decreasing concentrations of H151 (3.6, 1.8 and 0.9 μM) for 48 h, and IL-6 levels in supernatants were determined by ELISA. IL-6 levels were normalized to the “CPT only” condition and are shown as percentages. Data shown are averaged from three independent experiments in biological replicate (±s.e.m. and ordinary one-way ANOVA with Dunnett’s multiple comparison tests to the “CPT only” condition). **(D)** MG-63 and HOS were treated with CPT with or without 3.6 μM H151 for 48 h, and cell lysates were processed for RNA purification and RT-qPCR analyses. *IL-6* levels were reported relative to *18S* expression and divided further by the mean of the NT condition. Data shown are averaged from three independent experiments (±s.e.m. and ordinary one-way ANOVA with Dunnett’s multiple comparison tests to the “CPT only” condition). **(E)** MG-63 and HOS were treated with CPT with or without 200 nM WEHI-122 for 48 h and IL-6 levels in supernatants were determined by ELISA. IL-6 levels were normalized to the “CPT only” condition and are shown as percentages. Data shown are averaged from three (MG-63) or two (HOS) independent experiments in biological replicate (±s.e.m. and Mann-Whitney U tests are shown). **(F, G)** MG-63 were treated overnight with 100 nM GSK#3, with or without 3.6 μM H151 **(F)** or 200 nM WEHI-112 **(G)**, and IL-6 levels in supernatants were determined by ELISA. IL-6 levels were normalized to the “GSK only” condition and are shown as percentages. Data shown are averaged from three **(F)** or two **(G)** independent experiments in biological replicate (±s.e.m. and Mann-Whitney U tests are shown). **(H, I, J)** Wild-type (WT) **(H)**, *cGAS*-deficient **(I)** and *STING*-deficient **(J)** HaCaT cells were treated with 0.2 μM CPT in the presence of decreasing amounts of H151 (3.6, 1.8 or 0.9 μM) **(H)** or 3.6 μM **(I)** for 24 h, and IL-6 levels in supernatants were determined by ELISA. **(J)** Cells were treated with poly(I:C) [p(I:C)] at 1 μg/ ml, where indicated. IL-6 levels were normalized to the “CPT only” **(H, I)** or “p(I:C)” **(J)** condition and are shown as percentages. Data shown are averaged from two **(I)** or three **(H, J)** independent experiments in biological replicate [±s.e.m. and ordinary one-way ANOVA with Dunnett’s multiple comparison tests to the “CPT only” condition **(H)**, and Mann-Whitney U tests are shown **(I, J)**]. **(K)** MG-63 and HOS were transfected with 10 nM of the indicated siRNAs for 24 or 48 h, respectively, prior to CPT treatment for 48 or 24 h, respectively, and IL-6 levels in supernatants were determined by ELISA. IL-6 levels were normalized to the “CPT + siNC5” condition and are shown as percentages (±s.e.m. and ordinary one-way ANOVA with Dunnett’s multiple comparison tests to the “CPT + siNC5” condition). *p≤0.05, **p≤0.01, ***p≤0.001, ****p≤0.0001 and “ns” is non-significant.

This concurrent induction of *Il-6*, *Rsad2* and *Ifit1* by CPT in TC-1 cells prompted us to broadly assess whether such convergent induction of the NF-κB and IRF3 branches was a frequent response to DNA damage in cancer cells. For this purpose, we relied on a published dataset comparing the time-dependent transcriptional responses of cancer cells from the NCI60 panel, treated with several genotoxic agents (Monks et al., 2018). Forty-two cell lines in this panel significantly expressed *STING* based on the Cancer Cell Line Encyclopedia (Barretina et al., 2012), and were used for our *in silico* studies (Supplementary Table S1). Transcriptional analyses of *IL-6*, *RSAD2, IFIT1* and *IFNB1* following treatment with the CPT analogue topotecan (Top) suggested that 15 and 21 out of 42 human cancer cell lines expressing *STING* showed increased *IL-6* expression >2 fold after 6 and 24 h Top treatment, respectively (Figure 2A and Supplementary Table S1).

Critically, the induction of *IFIT1/RSAD2* and *IFNB1* was mostly divergent from that of *IL-6*, while being more restricted. As an example, 14/42 cell lines showed >2-fold increase in *IFIT1* expression at 24 h, but only five also displayed increased *IL-6* levels (Figure 2A). A similar observation was made with Doxorubicin (Dox)-driven topoisomerase 2 inhibition; albeit some of the cells that induced *IL-6* > 2 fold differed from those treated with Topotecan. Nonetheless, Dox treatment induced *IL-6* in 23/35 cells lines at 24 h with a 2-fold threshold, versus 16/35 for *IFIT1*—with only five cell lines showing increases in both genes (Figure 2A). Collectively, these analyses revealed that while *IL-6* was rapidly induced in 50% of cancer cells by DNA damage, this induction was often independent of that of ISGs.

### Inhibition of STING Palmitoylation Does Not Reduce IL-6 in MG-63 and SK-OV-3 Cells

To confirm the potential involvement of STING signaling in this rapid *IL-6* production upon DNA damage, we selected a subset of five cancer cell lines from this panel to which we had access (BT-549, HS-578T, MDA-MB-231, PC-3 and SK-OV-3 cells), that exhibited various profiles of *IL-6*/ISGs responses. For example, SK-OV-3 cells induced high amounts of *IL-6* but not ISG, while BT-549 cells robustly induced both *IL-6*/ISG responses (Figure 2A). MDA-MB-231 and HS-578T cells had more variable responses to Dox and Top but did induce *IL-6* and *IFIT1* >2 fold with Top, while PC3 displayed a stronger *IFIT1* induction than IL-6 with Top. We also tested two *STING*-expressing osteosarcoma lines we had previously found to produce IL-6 upon CPT treatment (HOS and MG-63 cells).

Low-dose CPT increased IL-6 production that was significantly inhibited by H151 in five cell lines (BT-549, HS-578T, MDA-MB-231, PC-3 and HOS cells), independent of increased cell death, supporting a direct contribution of canonical STING signaling in the pro-inflammatory response to CPT in these cells (Figure 2B; Supplementary Figures S1A,B). This aligned with the detection of *IFIT1*/ISG induction upon genotoxic treatment in our transcriptional analyses for BT-549, HS-578T, MDA-MB-231 and PC-3 cells (Figure 2A). Conversely, H151 failed to significantly reduce IL-6 production in MG-63 and SK-OV-3 cells (Figure 2B). Consistently with this, pharmacological inhibition of canonical STING or TBK1 failed to reduce the CPT-driven IL-6 induction at the mRNA and protein levels in MG-63, while it did in HOS cells (Figures 2C–E). MG-63, however, did produce IL-6 in response to a human synthetic STING agonist (referred to as GSK#3 herein - (Ramanjulu et al., 2018)), and this was significantly reduced by H151 or TBK1 inhibition (Figures 2F,G), confirming the capacity of MG-63 cells to also produce IL-6 through canonical STING signaling.

### Pharmacological Inhibition of STING Palmitoylation Does Not Impact Non-Canonical STING Signaling

Non-canonical STING signaling does not require translocation from the ER to the Golgi and as such is not impacted by TBK1 inhibition (Dunphy et al., 2018). Since STING palmitoylation occurs at the Golgi, we speculated that the lack of inhibitory activity of H151 in MG-63 cells could relate to non-canonical STING signaling being at play in these cells upon CPT treatment. We first confirmed that H151 could not inhibit CPT-driven IL-6 production stemming from non-canonical STING signaling in wild-type and *cGAS*-deficient HaCaT cells (Figures 2H,I, Material and Methods, and Supplementary Figures S1A,S2). Importantly, *STING* deficiency entirely abolished CPT-driven IL-6 production in HaCaT cells, confirming the reliance on STING for this non-canonical response (Figure 2J) (Dunphy et al., 2018). In agreement with this, RNA interference mediated down-regulation of STING significantly decreased CPT-driven IL-6 production in both MG-63 and HOS cells, demonstrating the dependence on STING in both cell lines (Figure 2K; Supplementary Figures S1C,D). Collectively, these results demonstrated that the inhibitory activity of H151 was limited to canonical STING signaling and supported the engagement of non-canonical STING signaling upon genotoxic DNA damage in select cancer cell lines.

### Inhibition of Downstream MAP Kinases Broadly Suppresses CPT-Driven IL-6

**FIGURE 3:**
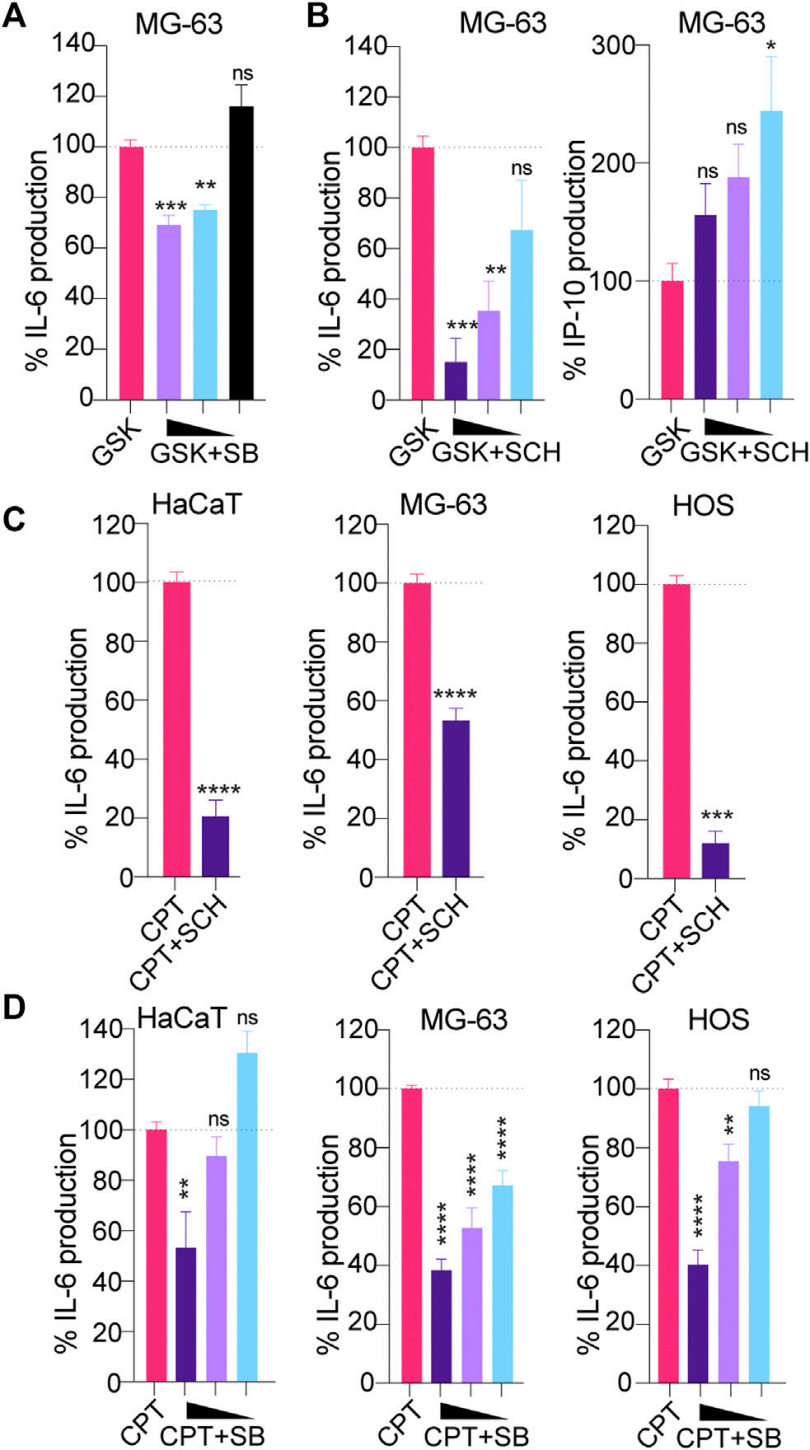
**(A, B)** MG-63 were treated overnight with 100 nM of the STING agonist GSK#3 with or without decreasing concentrations of the p38 inhibitor SB202190 [SB] (at 3, 1.5 and 0.75 μM) **(A)** or decreasing concentrations of the ERK1/2 inhibitor SCH772984 [SCH] (at 1.25, 0.63 and 0.313 μM) **(B)**, and IL-6/IP-10 levels in supernatants were determined by ELISA. IL-6 and IP-10 levels were normalized to the “GSK only” condition and are shown as percentages. **(A, B)** Data shown are averaged from two independent experiments in biological replicate (±s.e.m. and ordinary one-way ANOVA with Dunnett’s multiple comparison tests to the “GSK only” condition). **(C)** HaCaT WT, MG-63 and HOS were treated with CPT for 24 (HaCaT) or 48 h (MG-63 and HOS), with or without 1 μM SCH, and IL-6 levels in supernatants were determined by ELISA. IL-6 levels were normalized to the “CPT only” condition and are shown as percentages. Data shown are averaged from a minimum of three independent experiments in biological replicate (±s.e.m. and Mann-Whitney U tests are shown). **(D)** HaCaT WT, MG-63 and HOS were treated with CPT for 24 (HaCaT) or 48 h (MG-63 and HOS), with or without decreasing concentrations of SB (at 3, 1.5 and 0.75 μM) and IL-6 levels in supernatants were determined by ELISA. IL-6 levels were normalized to the “CPT only” condition and are shown as percentages. Data shown are averaged from a minimum of three independent experiments in biological replicate (±s.e.m. and ordinary one-way ANOVA with Dunnett’s multiple comparison tests to the “CPT only” condition). *p≤0.05, **p≤0.01, ***p≤0.001, ****p≤0.0001 and “ns” is non-significant.

The lack of activity of H151 on non-canonical STING signaling led us to investigate whether targeting of downstream mediators of NF-κB signaling could help broadly dampen CPT-driven IL-6 production, independent of the type of STING signaling engaged. Non-canonical STING has been shown to rely on TRAF6 activity (Dunphy et al., 2018). While the signaling components operating downstream of TRAF6 to control STING-driven IL-6 have not been characterized to date, we posited a role for mitogen-activated protein kinases (p38 and ERK1/2) based on their known involvement in DNA-damage responses and control of IL-6 expression (Craig et al., 2000; Phong et al., 2010; Wei et al., 2011; Dainichi et al., 2019). Inhibition of ERK1/2 with SCH772984 (Morris et al., 2013) and p38 with SB202190 were initially assessed with dose responses on canonical STING signaling induced with the GSK#3 STING agonist in MG-63 cells (Figures 3A,B). p38 and ERK1/2 inhibition both significantly reduced STING-driven IL-6 production in these cells (Figures 3A,B), although the effect was more potent with ERK1/2 inhibition. In agreement with a selective effect on NF-κB signaling downstream of canonical STING signaling, ERK1/2 inhibition did not reduce but rather increased IP-10 production upon GSK#3 stimulation - consistent with the prior findings that ERK1/2 inhibit type-I IFN production (Figure 3B) (Janovec et al., 2018).

We next studied the effect of p38 and ERK1/2 inhibition in MG-63/HaCaT cells (non-canonical STING) and HOS cells (canonical STING) treated with low-dose CPT. Both inhibitors lead to a significant reduction of CPT-driven IL-6 in the three cell models, without impacting further cell viability (Figures 3C,D, Supplementary Figure S1A), suggesting that they may be suitable to control the production of pro-tumorigenic factors upon DNA damage.

### Pharmacological Inhibition of STING Palmitoylation can Lead to Increased Cancer Cell Growth

**FIGURE 4:**
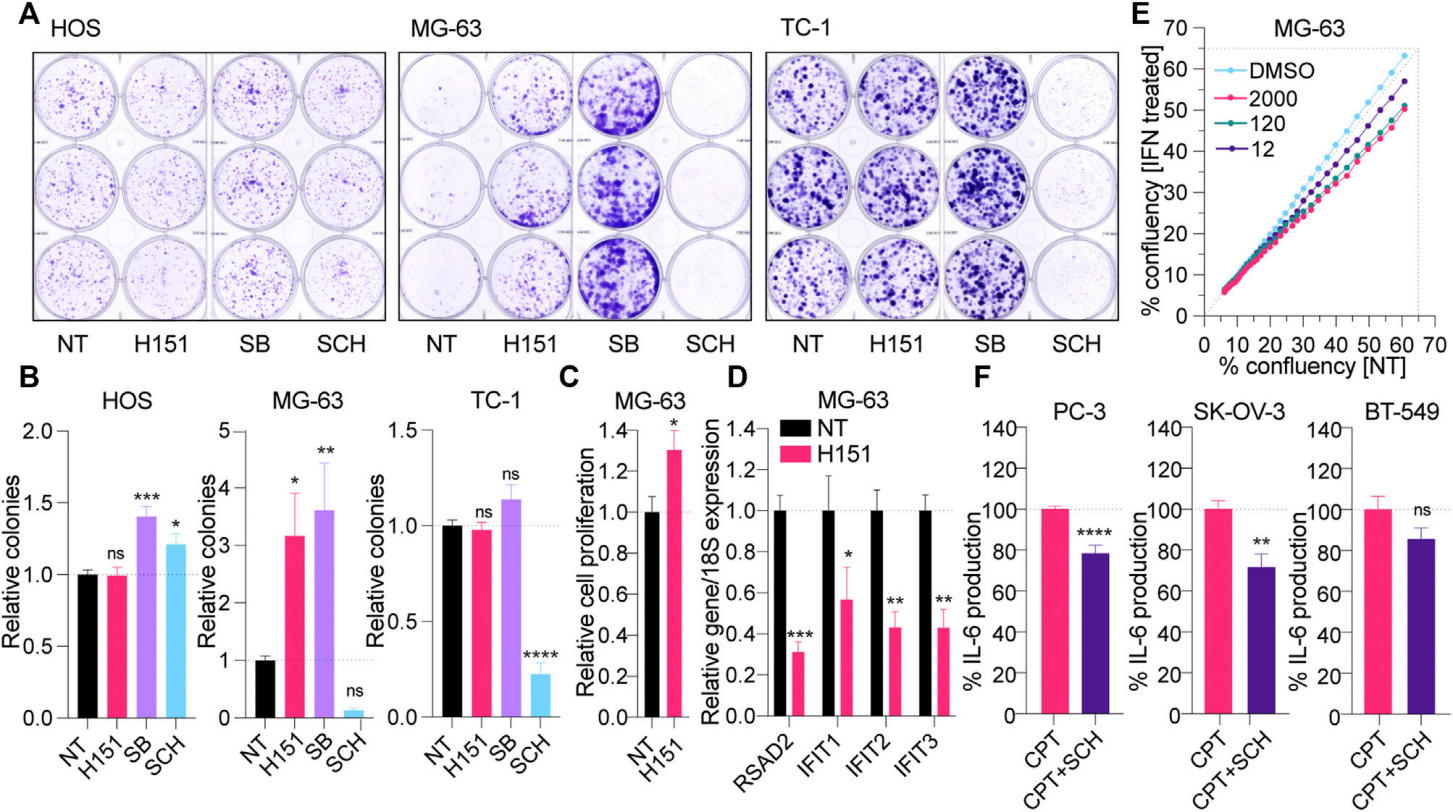
**(A, B)** HOS, MG-63 and TC-1 cells were plated at low density and treated with 3.6 μM H151, 3 μM of the p38 inhibitor SB202190 [SB] or 1 μM of the ERK1/2 inhibitor SCH772984 [SCH] for 10, 12, and 7 days, respectively (see Materials and Methods). The clones formed were stained with crystal violet after fixing **(A)** and counted manually **(B)**. **(B)** The number of colonies were reported to the NT condition. The data shown are representative **(A)** or averaged **(B)** from three independent experiments (in biological triplicate) (±s.e.m. and ordinary one-way ANOVA with Dunnett’s multiple comparison tests to the “NT” condition). **(C)** Proliferation of MG-63 cells treated with 3.6 μM H151 was measured by xCELLigence real-time monitoring over 48 h of treatment. Cell proliferation slopes were calculated and normalized to the NT condition. Data shown are averaged from three independent experiments in biological triplicate (±s.e.m. and Mann-Whitney U tests are shown). **(D)** MG-63 cells were treated or not with 3.6 μM H151 for 48 h, and cell lysates were processed for RNA purification and RT-qPCR analyses. Expression of the indicated genes was reported relative to 18S expression. Data shown are averaged from three independent experiments in biological duplicate and normalized to the NT condition (±s.e.m. and ordinary two-way ANOVA with Sidak’s multiple comparison tests). **(E)** Relative confluency of MG-63 cells with or without (NT) increasing concentrations of recombinant IFN (12, 120, 2000 IU/ ml) was assessed over 48 h with Incucyte. Data shown are averaged from 6 wells per condition, and trends are representative of three independent experiments. **(F)** PC-3, SK-OV-3 and BT-549 cells were treated with CPT for 24 h with or without 1 μM SCH, and IL-6 levels in supernatants were determined by ELISA. IL-6 levels were normalized to the “CPT only” condition and are shown as percentages. Data shown are averaged from a minimum of three independent experiments in biological replicate (±s.e.m. and Mann-Whitney U tests are shown). *p≤0.05, **p≤0.01, ***p≤0.001, ****p≤0.0001 and “ns” is non-significant.

Having demonstrated the capacity of H151 to block CPT-induced canonical STING signaling in selected cell lines, we next assessed its impact on cancer cell proliferation, independent of DNA damage, compared to p38 and ERK1/2 inhibition. MG-63, HOS, and TC-1 cells were grown in the continuous presence of H151, SB202190 or SCH772984 for 7–12 days in clonogenic assays. Surprisingly, H151 and SB202190 robustly increased clone formation in MG-63 cells (Figures 4A,B). This positive effect of H151 on clone formation was limited to MG-63 cells and reflected by increased growth curves (Figure 4C). However, SB202190 also potentiated the growth of HOS cells and, to a lesser extent, TC-1 cells (Figures 4A,B). Conversely, ERK1/2 inhibition with SCH772984 strongly limited the expansion of MG-63 and TC-1 cells, and modestly impacted that of HOS cells (Figures 4A,B). Having previously shown that MG-63 had a functional cGAS-STING response (Valentin et al., 2021), we reasoned that H151 may block canonical STING signaling basally engaged in these cells, normally restraining their growth. Accordingly, the basal expression of several ISGs (*RSAD2*, *IFIT1*, *IFIT2*, *IFIT3*) was significantly decreased by H151 treatment in MG-63 cells (Figure 4D). In addition, treatment of MG-63 cells with increasing amounts of type-I IFN significantly decreased the growth of the cells (Figure 4E), supporting the concept that H151 increased cell proliferation through the inhibition of constitutive anti-proliferative interferon signaling.

Finally, since it appeared to limit CPT-driven inflammation from both canonical and non-canonical STING signaling, without promoting cancer cell proliferation, we also tested the effect of ERK1/2 inhibition with SCH772984 on CPT-treated PC-3, SK-OV-3 and BT-549 cells (Figure 4F). Although less potent than in the other cells, SCH772984 significantly reduced CPT-driven IL-6 in PC-3 and SK-OV-3 cells, supporting its broad anti-inflammatory effect independent of how STING is activated (noting that there was no significant effect of SCH772984 on cell viability–Supplementary Figure S1E). Nonetheless, SCH772984 did not significantly reduce IL-6 production in BT-549.

## Discussion

Well before its description as a selective agonist of murine Sting (Gao et al., 2013), the small molecule 5,6-dimethylxanthenone-4-acetic acid (DMXAA, or vadimezan) had been characterized as a strong anti-cancer drug that potentiated anti-cancer activities promoted by radio and chemotherapies in syngeneic murine cancer models (recently reviewed in (Le Naour et al., 2020)). Accordingly, several human STING agonists have been developed in recent years by academic and pharmaceutical industry laboratories (Ramanjulu et al., 2018; Chin et al., 2020; Pan et al., 2020), and clinical trials are underway to assess their efficacy against cancers in combination with immune checkpoint inhibitors (Le Naour et al., 2020). While it is clear that STING activation of the immune-cell compartment of the tumor microenvironment can have strong anti-tumoral activities, owing to production of anti-proliferative IFN-β (Parker et al., 2016) and the ensuing recruitment of CD8+T cells (Diamond et al., 2011), the cell-intrinsic role of STING signaling on the growth of cancer cells remains poorly defined.

In the current work, we investigated the cell-intrinsic effects of genotoxic DNA damage on STING signaling in human cancer cells. Our analyses of a dataset of 42 STING-expressing cancer cell lines demonstrated the frequent induction of IL-6 upon topoisomerase 1 and 2 inhibition in ≥ 50% of the cells, often independent of a marked ISG response. As such, 8/21 cells lines displaying *IL-6* increased with CPT ≥ two fold failed to show a significant induction of *IFIT1/RSAD2* or *IFNB1* at this threshold, indicating a preferential engagement of the NF-κB branch over that of IRF3 in a third of the cell lines. Although noticeable variations of *IL-6* induction existed for select cell lines between topoisomerase 1 and 2 inhibition, 16/21 cell lines responsive to Top also induced *IL-6* with Dox, often independently of ISG signatures. Nonetheless, 29/42 cell lines displayed increased induction of one of the 3 ISGs considered with either Dox or Top treatment, against 27/42 for *IL-6* induction. This confirms that both NF-κB and IRF3 branches are frequently engaged in cancer cells upon genotoxic treatment.

Critically, we showed that IL-6 induced by DNA damage was partially dependent on STING signaling in all the cell lines we tested–as revealed by a significant decrease in IL-6 production with pharmacological inhibition or down-regulation of *STING* expression. Given that up to 85% (819/934) of the cancer cell lines in the Cancer Cell Line Encyclopedia expressed STING, we speculate that STING-dependent IL-6 induction in response to DNA damage is very frequent in cancer cells. This aligns with the literature supporting that IL-6 and its activation of STAT3 counteracts the effects of radio- and chemotherapy in many cancers (Yang et al., 2020). It remains possible, however, that CPT-driven IL-6 production in select cancer cells is independent of STING, and reliant on alternative pathways involving other innate immune sensors detecting DNA damage from the nucleus or the mitochondria (Burleigh et al., 2020; Tigano et al., 2021). While warranting further studies in larger datasets of cancer cells, this constitutes, to our knowledge, the first direct evidence that cell-intrinsic canonical STING signaling frequently contributes to the production of pro-tumorigenic IL-6 upon DNA damage in cancer cells.

The recent study by Dunphy *et al.* suggested the existence of a cGAS-independent, non-canonical STING signaling, activated upon DNA damage with the topoisomerase 2 inhibitor Dox in human immortalized and primary keratinocytes (Dunphy et al., 2018). Although the study did not define whether this pathway was frequently invoked upon DNA damage in cancer cells (beyond the case of PMA-differentiated THP-1 cells), it is noteworthy that this alternative STING pathway favored the activation of NF-κB driven pro-inflammatory factors such IL-6, with limited IRF3 signaling (Dunphy et al., 2018).

Here we confirmed the observation from Dunphy *et al.* that HaCaT cells lacking *cGAS* can produce IL-6 upon DNA damage, in a *STING*-dependent manner (Dunphy et al., 2018). In support of the concept of a non-canonical STING signaling pathway, we demonstrated that pharmacological inhibition of STING palmitoylation did not impact CPT-driven IL-6 in these cells. Critically, we provide evidence that the occurrence of non-canonical STING signaling is not limited to keratinocytes, and that it can also be activated by DNA damage in cancer cells such as MG-63 cells. While IL-6 production was dependent on *STING* expression in MG-63 cells, pharmacological inhibition of canonical STING/TBK1 signaling did not reduce CPT-driven IL-6 in these cells. Although additional experiments would be required to confirm that the *STING*-dependent responses to DNA damage seen in HaCaT operate the same way in MG-63 cells, the hallmarks of the responses in both cell lines support the concept that they share key similarities. How frequently this non-canonical STING signaling is engaged in human cancers remains to be determined, but the fact that it can be engaged independently of cGAS suggests that it could be relatively common. For example, analyses of TCGA datasets indicate that >30% of high expressing STING lung adenocarcinoma or testicular cancer tumors have low cGAS expression (Supplementary Table S2).

Importantly, albeit failing to respond to H151 inhibition, MG-63 and HaCaT cells both responded to transfected DNA through canonical cGAS-STING signaling (Supplementary Figure S2) (Valentin et al., 2021). Perhaps most surprisingly, we demonstrated that prolonged exposure to H151 increased the growth of MG-63 cells, concurrently with a significant decrease of a basal ISG signature (noting that MG-63 cells are known to produce high levels of type-I IFN) (Billiau et al., 1977). These observations support a basal anti-proliferative activity of STING in MG-63 cells, through the IRF3/IFN arm of the pathway, supported by the reduced growth of the cells cultured in the presence of type-I IFN. This points to the capacity of MG-63 cells to rapidly switch between steady-state canonical STING signaling, most likely resulting from low levels of cytoplasmic DNA, to non-canonical STING signaling activated by acute DNA damage.

The results collectively obtained in MG-63 cells crystalize the duality of the pathway in cancer cells, which can rapidly shift from anti-proliferative to pro-tumorigenic in the context of DNA damage. Given how frequently rapid induction of *IL-6* was observed in cancer cells, the current concept that pro-tumorigenic activities of the pathway would be limited to its chronic engagement clearly needs revision (Decout et al., 2021). These findings are also important to our understanding of how to best apply STING agonists in cancer immunotherapy involving DNA damage, since IL-6 was found to inhibit the anti-tumoral effects of STING activation *in vivo* (Suter et al., 2021).

With the aim of inhibiting the pro-tumorigenic NF-κB branch of STING signaling, but retaining that of IRF3/IFN-β, we discovered that inhibition of ERK1/2 was able to reduce IL-6 production upon canonical and non-canonical STING activation. Critically, ERK1/2 inhibition did not compromise the IRF3 branch of STING signaling, as seen with preserved IP-10 levels in MG-63 cells treated with a human STING agonist. Accordingly, in addition to its own anti-cancer activities (Kidger et al., 2018), pharmacological ERK1/2 inhibition may be a viable strategy to broadly decrease IL-6 production upon DNA damage, while retaining the anti-proliferative effects of the pathway, seen in MG-63 cells. Although further studies are warranted, this is the first description, to our knowledge, that ERK1/2 participate in the production of pro-inflammatory factors downstream of STING. Note that ERK1/2 phosphorylation has been reported in mouse embryonic fibroblasts stimulated with DMXAA (Abe and Barber, 2014). Nevertheless, ERK1/2 inhibition may not universally limit IL-6 production driven by DNA damage in cells where the IRF3 branch of STING signaling dominates the response to DNA damage, as suggested by our results in BT-549 cells.

In conclusion, we demonstrate here that STING is an important contributor to the rapid IL-6 production frequently seen upon DNA damage in cancer cells. Our results collectively indicate that targeting of signaling components operating downstream of STING to modulate NF-κB activity may be more useful than direct STING inhibitors to help prevent production of pro-tumorigenic factors such as IL-6. We propose that pharmacological targeting of ERK1/2, which is already investigated in cancer patients with oncogenic RAS-dependent tumors (Lu et al., 2020), may also help attenuate the resistance to radio- and chemotherapy treatments mediated in part by STING-dependent pro-inflammatory factors, while retaining the anti-tumor activity of the IRF3/IFN-β branch of the pathway.

## Data Availability

The original contributions presented in the study are included in the article/Supplementary Material, further inquiries can be directed to the corresponding authors.
