# Supplementary material for: Pharmacological Targeting of STING-Dependent IL-6 Production in Cancer Cells
Source: Front Cell Dev Biol. 2022 Jan 11;9:709618. doi: 10.3389/fcell.2021.709618 (PMC8787270; doi:10.3389/fcell.2021.709618)
Supplement: Supplementary file 1 [file DataSheet1.PDF]

# Supplementary Material

## 1 Supplementary Materials and Methods

### Cell culture

Camptothecin (CPT - Sigma # C9911 – resuspended in DMSO prior to dilution in medium) was used at the following concentrations to induce DNA damage: 0.1 µM (MG-63, SK-OV-3, BT-549), 0.2 µM (HOS, HaCaT) and 0.5 µM (PC-3, MDA-MB-231, HS-578T, TC-1). Inhibitors H151 (Cayman, #25857), SB202190 (Sigma, #S7067) and SCH772984 (Cayman, #19166), were resuspended in DMSO. Inhibition of CPT-stimulation was carried out with <0.5% DMSO per well. The human STING agonist (compound #3 from (Ramanjulu et al., 2018), referred to as GSK#3 herein – kind gift from Cancer Therapeutics CRC, Australia) was used at 100 nM. The TBK1 inhibitor, WEHI-112 (kind gift from D. De Nardo), was used at 200 nM. Poly(I:C) (Invivogen, #tlrl-pic) was used at 1 µg/ml. Recombinant human IFN-α (IntronA, SP Europe) was used at indicated doses.

Cell viability was assessed by adding 1X fresh resazurin solution to the wells (10X solution made up with 7 mg resazurin [Sigma, # R7017] dissolved in 3.5 ml PBS, filter sterilized at 0.2 µM) for ~4 h, prior to reading with a Fluostar OPTIMA (BMG LABTECH) plate-reader (fluorescence: excitation 535 nm, emission 590); wells with medium only and 1X resazurin were used as blanks.

For determination of the clone counts shown in Figure 4B, the round-spheroid colonies consisting of ≥ 50 cells were counted manually from 3 replicate wells per independent experiment.

### RNA interference

12,000 MG-63 or HOS cells per well (96-well plate) were reverse transfected with 10 nM of the indicated siRNA (1.35 µl lipofectamine 2000 / 1.5 µl of each siRNA at 4 µM for 3 wells with 200 µl final volume) for 24 h (MG-63) or 48 h (HOS), before being washed and treated with CPT for 24 (HOS) or 48 h (MG-63). All siRNAs were synthesized as pre-annealed Dicer substrates by Integrated DNA Technologies (r = RNA base and Uppercase = DNA base). Sequences used were: human siSTING-1 (hs.Ri.TMEM173.13.1-SEQ1 rArArU rCrArG rCrArU rUrArC rArArC rArArC rCrUrG rCrUA C; hs.Ri.TMEM173.13.1-SEQ2 rGrUrA rGrCrA rGrGrU rUrGrU rUrGrU rArArU rGrCrU rGrArU rUrGrU); human siSTING-4 (hs.Ri.TMEM173.13.4-SEQ1 rGrCrA rUrUrA rCrArA rCrArA rCrCrU rGrCrU rArCrG rGrGG T; hs.Ri.TMEM173.13.4-SEQ2 rArCrC rCrCrG rUrArG rCrArG rGrUrU rGrUrU rGrUrA rArUrG rCrUrG). The non-targeting siNC5 siRNA was used as control (Sarvestani et al., 2014). Validation of the down-regulation was carried out after 48 h transfection by Western blotting, as previously described (Pepin et al., 2016). Protein detection was carried out using 1:1000 rabbit α-STING [D2P2F] Rabbit mAb, (Cell Signaling Technology, #13647) or rat monoclonal anti-tubulin (YL1/2 | ab6160, Abcam). Finally, conjugated secondary antibodies with IRdye800 (Rockland) were used to image the proteins at 800 nm with a ChemicDoc scanner (Bio-Rad).

### xCELLigence real-time cell analyses

A total of 10,000 MG-63 cells were monitored in real-time using the xCELLigence Single Plate analyzer (Agilent, Model W380) with a 96-well format electronic microtiter plate (E-plate 96). Cell

proliferation was monitored over a period of 72 h, the first 24 h being used to measure cellular adherence and attachment efficiency to the E-plate without any added compounds, for normalization of cellular index reads. The following 48 h were used to measure MG-63 cell proliferation every 60 minutes in the presence of 3.6 μM H151 in triplicate wells.

**Cell proliferation analyses**

For Figure 4E, MG-63 cells were seeded into clear-walled 96-well plates (Nunc) at a cell density of 3 × $10^3$ cells per well and allowed to attach overnight. Drug treatments were added as indicated and wells were imaged every 2 h over 96 h using the Incucyte S3 (Essen BioScience, Ann Arbor, MI, USA). Image analysis of cell proliferation was performed using the Incucyte Zoom software package. Proliferation curves were generated using GraphPad Prism V9 software.

**Detection of cytokines**

Human IP-10 and IL-6 levels were measured using supernatants from the different cultures and were quantified using IP-10 (BD Biosciences, #550926) or IL-6 (BD Biosciences, # 555220, and Thermo Fisher Scientific, #88-7066-88) ELISA kits respectively; mouse IP-10 and IL-6 levels were measured using Mouse CXCL10/IP-10/CRG-2 Duo Set ELISA (R&D systems, #Dy466) and IL-6 (BD Biosciences, # 555240) ELISA kits respectively, according to the manufacturers' protocols. Tetramethylbenzidine substrate (Thermo Fisher Scientific) was used for quantification of the cytokines on a Fluostar OPTIMA (BMG LABTECH) plate-reader.

**mRNA reverse transcription quantitative real-time PCR (RT-qPCR)**

Total RNA was purified from cells using the ISOLATE II RNA Mini Kit (Bioline). Random hexamer cDNA was synthesized from isolated RNA using the High-Capacity cDNA Archive kit (Thermo Fisher Scientific) according to the manufacturer's instructions. RT-qPCR was carried out with the Power SYBR Green Master Mix (Thermo Fisher Scientific) on the QuantStudio 6 RT-PCR system (Thermo Fisher Scientific). Each PCR was carried out in technical duplicate and human or mouse 18S was used as the reference gene. Each amplicon was gel-purified and used to generate a standard curve for the quantification of gene expression (used in each run). Melting curves were used in each run to confirm specificity of amplification. The primers used were the following: Human 18S: h18S-FWD CGGCTACCACATCCAAGGAA; h18S-REV GCTGGAATTACCGCGGCT; Human IFIT1: IFIT1-FWD TCACCAGATAGGGCTTTGCT; hIFIT1-REV CACCTCAAATGTGGGCTTTT; Human IFIT2: IFIT2-RT-FWD TTATTGGTGGCAGAAGAGGAAG; IFIT2-RT-REV CCTCCATCAAGTTCCAGGTG; hIFIT3: hIFIT3-FWD CATAAAAGCACAGACCTAACAGC; hIFIT3-REV CAGGGAATTCTTGGTGACCTC; Human RSAD2: hRSAD2-RT-FWD TGGTGAGGTTCTGCAAAGTAG; hRSAD2-RT-REV GTCACAGGAGATAGCGAGAATG; hIL6-RT-FWD CACCGGGAACGAAAGAGAAG; hIL6-RT-REV TCATAGCTGGGCTCCTGGAG; Mouse 18s: Rn18s-FWD GTAACCCGTTGAACCCCATT; Rn18s-REV CCATCCAATCGGTAGTAGCG; Mouse Rsad2: Rsad2-FWD CTGTGCGCTGGAAGGTTT; Rsad2-REV ATTCAGGCACCAAACAGGAC; Mouse Ifit1: Ifit1-RT-FWD GAGAGTCAAGGCAGGTTTCT; Ifit1-RT-REV TCTCACTTCCAAATCAGGTATGT; Mouse Il6: Il6-RT-FWD: ATGGATGCTACCAAACTGGAT; Il6-RT-REV TGAAGGACTCTGGCTTTGTCT.

**CRISPR gene editing of HaCaT cells**

Cas9 ribonucleoprotein (RNP) assembly was performed following the manufacturer's recommendations (IDT), as per our previous publication (Ferrand et al., 2018). The crRNA guide sequences employed were GCUGGGACUGCUGUUAAACGGUUUUAGAGCUAUGCU (human STING) and AGACUCGGUGGGAUCCAUCGGUUUUAGAGCUAUGCU (human cGAS) - the *STING* and *cGAS*-specific protospacer elements are underlined. 120 pmol of crRNA:tracrRNA was mixed with 100 pmol of recombinant Cas9 protein (Alt-R® Cas9 Nuclease 3NLS) and set to incubate at room temperature for 15 minutes. Electroporation transfection of 500,000 HaCaT cells was performed using a mix of 90 µl of BTXPRESS electroporation buffer (BTX) and 10 µl of the Cas9 RNP complex in an AMAXA Nucleofactor II (Lonza). After 2 days recovery at 37°C, cloning by limiting dilution was performed and STING/cGAS deficiency was identified by functional assay with transfection of ISD70 (Pépin et al., 2020), and western blotting (antibodies: α-cGAS [D1D3G] Rabbit mAb, Cell Signaling Technology, #15102; and α-STING [D2P2F] Rabbit mAb, Cell Signaling Technology, #13647).

**Statistical analyses**

Statistical analyses were carried out using Prism 9 (GraphPad Software Inc.). Every experiment was repeated a minimum of two independent times, with biological duplicate or triplicate. One-way and two-way analyses of variance (ANOVA) were used when comparing groups of conditions, while two-tailed unpaired Mann-Whitney U tests were used when comparing pairs of conditions. Symbols used: * $P \leq 0.05$, ** $P \leq 0.01$, *** $P \leq 0.001$, **** $P \leq 0.0001$ and "ns" is non-significant.

## 2 Supplementary Figures

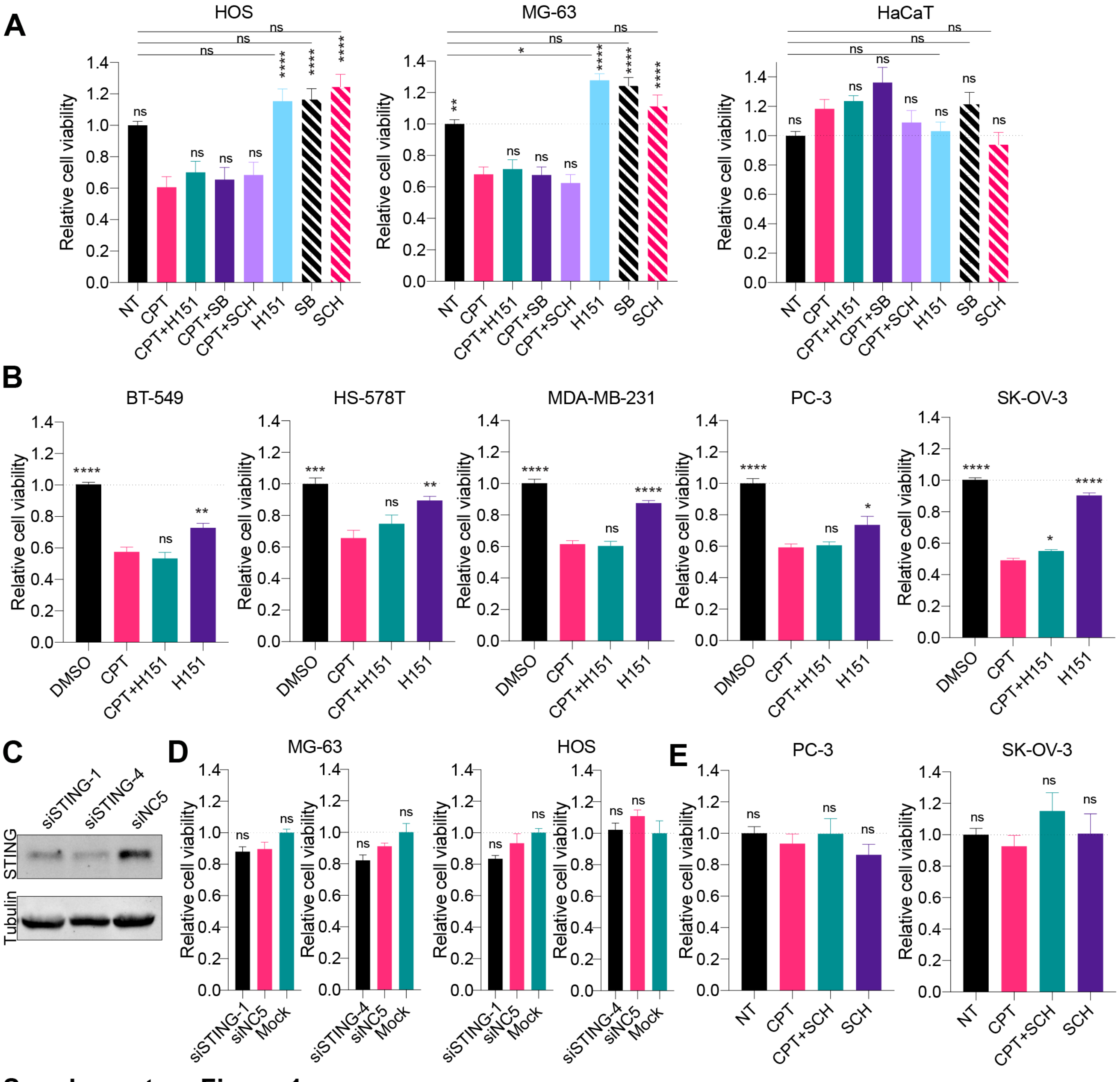


**Supplementary Figure 1**

**Supplementary Figure 1.** A and E) Cells were treated with CPT, with or without the indicated drugs for 24 (HaCaT, PC-3 and SK-OV-3 cells) or 48 h (HOS and MG-63 cells), prior to being assessed for cell viability using resazurin. Fluorescence levels were normalized to the “NT condition” after background correction with medium only. B) Cells were treated with CPT, with or without the indicated drugs for 24 h. Following partial removal of media for ELISA assays, the DNA-binding dye Hoechst 33342 was added to the wells for 30 min and cells were imaged and counted using the IN-Cell Analyzer 6500 HS high content imaging system. Data were normalized to the DMSO control condition.

C, D) MG-63 and HOS were transfected with 10 nM of the indicated siRNA for 48 h, respectively, prior to Western blot analyses (C) or cell viability using resazurin (D). C) The blots shown are representative of 2 and 3 independent experiments for siSTING-4 and siSTING-1, respectively. D) Fluorescence levels were normalized to the “Mock condition” (lipofectamine only) after background correction with medium only. A, B, D and E) Data shown are averaged from a minimum of two independent experiments in biological replicate (± s.e.m. and ordinary one-way ANOVA with or one-way ANOVA with Tukey’s multiple comparison tests to “CPT only” or as indicated [A], or Dunnett’s multiple comparison tests to the “CPT only” [B, E] or “siNC5” [D] conditions).

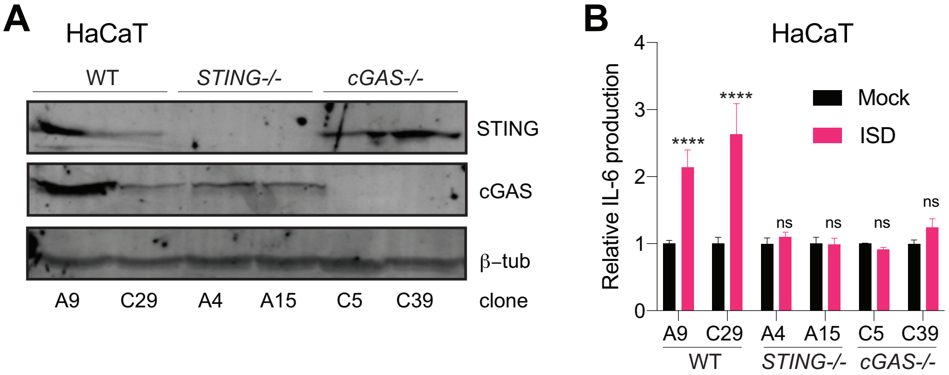


**Supplementary Figure 2.** A) HaCaT WT, *STING*- and *cGAS*-deficient clones generated by CRISPR/Cas9 were analyzed by Western blotting to confirm the effect of gene editing at the protein level. B) Indicated clones were transfected overnight with ISD70 or lipofectamine 2000 only (Mock) (as per (Pépin et al., 2020)). IL-6 levels in supernatants were determined by ELISA. IL-6 levels were normalized to the “Mock” condition for each clone. Data shown are averaged from two independent experiments in biological triplicate (± s.e.m. and ordinary two-way ANOVA with Sidak’s multiple comparison tests).

# 3 Supplementary Tables

**Cancer Cell Line Encyclopedia**

| Name | CGAS | TMEM173 |
|---|---|---|
| HCT-15, colon adenocarcinoma | 0.1 | 17 |
| SW620, colon adenocarcinoma | 0.1 | 7 |
| T-47D, invasive ductal carcinoma | 0.1 | 1 |
| HCT 116, colon carcinoma | 0.1 | 7 |
| U-251 MG, astrocytoma | 0.1 | 40 |
| OVCAR-3, high grade ovarian serous adenocarcinoma | 0.2 | 11 |
| SK-MEL-5, cutaneous melanoma | 0.2 | 1 |
| SK-OV-3, ovarian serous cystadenocarcinoma | 0.2 | 46 |
| UACC-257, melanoma | 0.3 | 43 |
| IGROV1, ovarian endometrioid adenocarcinoma | 0.9 | 7 |
| MDA-MB-468, breast adenocarcinoma | 2 | 53 |
| Malme-3M, melanoma | 2 | 9 |
| EKVX, non-small cell lung carcinoma | 2 | 58 |
| MCF7, invasive ductal carcinoma | 3 | 2 |
| OVCAR-4, high grade ovarian serous adenocarcinoma | 3 | 81 |
| NCI-H460, large cell lung carcinoma | 3 | 4 |
| A-498, renal cell carcinoma | 4 | 27 |
| SK-MEL-28, cutaneous melanoma | 5 | 22 |
| Hs 578T, invasive ductal carcinoma | 6 | 21 |
| Caki-1, clear cell renal carcinoma | 6 | 18 |
| PC-3, prostate carcinoma | 7 | 6 |
| UACC-62, melanoma | 7 | 20 |
| SF268, astrocytoma | 8 | 9 |
| HOP-92, non-small cell lung carcinoma | 9 | 43 |
| SF-295, glioblastoma | 10 | 21 |
| NCI-H226, small cell lung carcinoma | 11 | 14 |
| HT-29, rectosigmoid adenocarcinoma | 12 | 11 |
| HL-60, adult acute myeloid leukemia | 17 | 9 |
| BT-549, invasive ductal carcinoma | 19 | 5 |
| SNB75, glioblastoma | 20 | 36 |
| HOP-62, lung adenocarcinoma | 21 | 19 |
| NCI-H322, bronchoalveolar adenocarcinoma | 23 | 3 |
| LOX IMVI, amelanotic melanoma | 24 | 37 |
| NCI-H522, lung adenocarcinoma | 24 | 2 |
| DU 145, prostate carcinoma | 25 | 35 |
| SF539, gliosarcoma | 27 | 106 |
| RPMI 8226, multiple myeloma | 28 | 10 |
| MDA-MB-435S, amelanotic melanoma | 30 | 26 |
| MDA-MB-231, breast adenocarcinoma | 41 | 31 |
| NCI-H23, lung adenocarcinoma | 47 | 2 |
| OVCAR-8, high grade ovarian serous adenocarcinoma | 48 | 26 |
| UO-31, renal cell carcinoma | NA | 13 |

**NCI response to Topotecan**

| Name | 6h | 24h |
|---|---|---|
| HCT-15 | -0.54 | -0.09 |
| SW-620 | -0.21 | -0.11 |
| T-47D | 0.34 | -0.07 |
| HCT-116 | 0.41 | -0.50 |
| U251 | 1.05 | 0.91 |
| OVCAR-3 | -0.14 | 0.33 |
| SK-MEL-5 | -0.08 | -0.07 |
| SK-OV-3 | 2.28 | 2.77 |
| UACC-257 | -0.39 | -0.13 |
| IGR-OV1 | -0.40 | -0.05 |
| MDA-MB-468 | 0.04 | -0.06 |
| MALME-3M | 0.23 | 0.42 |
| EKVX | 0.26 | 0.50 |
| MCF7 | -0.23 | 0.29 |
| OVCAR-4 | -0.02 | 0.28 |
| NCI-H460 | 0.99 | 1.76 |
| A498 | -0.01 | 1.96 |
| SK-MEL-28 | -0.11 | -0.33 |
| HS-578T | 0.59 | 1.01 |
| CAKI-1 | 3.25 | 3.09 |
| PC-3 | -0.34 | 0.16 |
| UACC-62 | 0.05 | -0.41 |
| SF-268 | 1.47 | 1.96 |
| HOP-92 | 1.11 | 1.00 |
| SF-295 | 3.31 | 2.05 |
| NCI-H226 | -0.12 | 4.24 |
| HT29 | -0.23 | 0.75 |
| HL-60 | -0.12 | -0.59 |
| BT-549 | 1.23 | 2.68 |
| SNB-75 | 2.69 | 2.76 |
| HOP-62 | 1.07 | 1.27 |
| NCI-H322M | 0.21 | 0.53 |
| LOX | 0.27 | 0.63 |
| NCI-H522 | 0.41 | -0.04 |
| DU-145 | 0.26 | 0.39 |
| SF-539 | 1.44 | 3.25 |
| RPMI-8226 | -0.08 | 0.00 |
| MDA-MB-435 | 0.10 | -0.38 |
| MDA-MB-231 | 1.64 | 1.28 |
| NCI-H23 | -0.19 | -0.11 |
| OVCAR-8 | 4.72 | 3.51 |
| UO-31 | 2.63 | 1.47 |

**Supplementary Table 1.** Left panel: NCI-60 cell lines were sorted according to their level of STING expression as per RNA sequencing from the Cancer Cell Line Encyclopedia dataset (Barretina et al., 2012), analyzed through the Expression Atlas server (Papatheodorou et al., 2019). Only cell lines expressing STING with >1 CPM were used for our analyses (42 cell lines). Cell lines with <1 CPM cGAS expression (considered here to be negligible) are highlighted in blue. The $\log_2$ relative IL-6 expression in response to topotecan for each cell line at 6 and 24 h from (Monks et al., 2018), is given in the right panel.

| | Top 25% STING | Bottom 25% cGAS | % of low cGAS in high STING | Total samples |
|---|---|---|---|---|
| GDC TCGA Lung Adenocarcinoma (LUAD) | 148 | 52 | 35.1% | 585 |
| GDC TCGA Testicular Cancer (TGCT) | 40 | 13 | 32.5% | 156 |
| GDC TCGA Rectal Cancer (READ) | 44 | 14 | 31.8% | 177 |
| GDC TCGA Endometrioid Cancer (UCEC) | 146 | 38 | 26.0% | 583 |
| GDC TCGA Ovarian Cancer (OV) | 94 | 24 | 25.5% | 379 |
| GDC TCGA Acute Myeloid Leukemia | 37 | 9 | 24.3% | 151 |
| GDC TCGA Colon Cancer (COAD) | 130 | 29 | 22.3% | 512 |
| GDC TCGA Head and Neck Cancer (HNSC) | 136 | 30 | 22.1% | 546 |
| GDC TCGA Sarcoma (SARC) | 68 | 15 | 22.1% | 265 |
| GDC TCGA Pancreatic Cancer (PAAD) | 45 | 9 | 20.0% | 183 |
| GDC TCGA Cervical Cancer (CESC) | 78 | 15 | 19.2% | 309 |
| GDC TCGA Stomach Cancer (STAD) | 101 | 17 | 16.8% | 407 |
| GDC TCGA Kidney Papillary Cell Carcinoma (KIRP) | 80 | 13 | 16.3% | 321 |
| GDC TCGA Breast Cancer (BRCA) | 304 | 45 | 14.8% | 1217 |
| GDC TCGA Glioblastoma (GBM) | 44 | 6 | 13.6% | 173 |
| GDC TCGA Thyroid Cancer (THCA) | 142 | 15 | 10.6% | 568 |
| GDC TCGA Bladder Cancer (BLCA) | 107 | 11 | 10.3% | 430 |
| GDC TCGA Esophageal Cancer (ESCA) | 44 | 4 | 9.1% | 173 |
| GDC TCGA Lung Squamous Cell Carcinoma (LUSC) | 137 | 12 | 8.8% | 550 |
| GDC TCGA Prostate Cancer (PRAD) | 140 | 10 | 7.1% | 551 |
| GDC TCGA Kidney Clear Cell Carcinoma (KIRC) | 153 | 7 | 4.6% | 607 |
| GDC TCGA Pheochromocytoma & Paraganglioma (PCPG) | 46 | 2 | 4.3% | 186 |
| GDC TCGA Liver Cancer (LIHC) | 107 | 4 | 3.7% | 425 |
| GDC TCGA Lower Grade Glioma (LGG) | 132 | 4 | 3.0% | 529 |

**Supplementary Table 2.** Samples from the indicated cancers from the TCGA database were ranked according to STING and cGAS expression (into top and bottom quartiles), relying on the Xena platform (Goldman et al., 2020). For each cancer, the percentage of samples with the lowest cGAS levels was calculated among the highest STING-expressing ones. This calculation informs on the proportion of samples potentially lacking cGAS ,while expressing STING.

**References**

Barretina, J., Caponigro, G., Stransky, N., Venkatesan, K., Margolin, A.A., Kim, S., Wilson, C.J., Lehár, J., Kryukov, G.V., Sonkin, D., Reddy, A., Liu, M., Murray, L., Berger, M.F., Monahan, J.E., Morais, P., Meltzer, J., Korejwa, A., Jané-Valbuena, J., Mapa, F.A., Thibault, J., Bric-Furlong, E., Raman, P., Shipway, A., Engels, I.H., Cheng, J., Yu, G.K., Yu, J., Aspesi, P., De Silva, M., Jagtap, K., Jones, M.D., Wang, L., Hatton, C., Palescandolo, E., Gupta, S., Mahan, S., Sougnez, C., Onofrio, R.C., Liefeld, T., Macconaill, L., Winckler, W., Reich, M., Li, N., Mesirov, J.P., Gabriel, S.B., Getz, G., Ardlie, K., Chan, V., Myer, V.E., Weber, B.L., Porter, J., Warmuth, M., Finan, P., Harris, J.L., Meyerson, M., Golub, T.R., Morrissey, M.P., Sellers, W.R., Schlegel, R., and Garraway, L.A. (2012). The Cancer Cell Line Encyclopedia enables predictive modelling of anticancer drug sensitivity. *Nature* 483**,** 603-607.

Ferrand, J., Croft, N.P., Pépin, G., Diener, K.R., Wu, D., Mangan, N.E., Pedersen, J., Behlke, M.A., Hayball, J.D., Purcell, A.W., Ferrero, R.L., and Gantier, M.P. (2018). The Use of CRISPR/Cas9 Gene Editing to Confirm Congenic Contaminations in Host-Pathogen Interaction Studies. *Frontiers in Cellular and Infection Microbiology* 8.

Goldman, M.J., Craft, B., Hastie, M., Repecka, K., Mcdade, F., Kamath, A., Banerjee, A., Luo, Y., Rogers, D., Brooks, A.N., Zhu, J., and Haussler, D. (2020). Visualizing and interpreting cancer genomics data via the Xena platform. *Nat Biotechnol* 38**,** 675-678.

Monks, A., Zhao, Y., Hose, C., Hamed, H., Krushkal, J., Fang, J., Sonkin, D., Palmisano, A., Polley, E.C., Fogli, L.K., Konaté, M.M., Miller, S.B., Simpson, M.A., Voth, A.R., Li, M.-C., Harris, E., Wu, X., Connelly, J.W., Rapisarda, A., Teicher, B.A., Simon, R., and Doroshow, J.H. (2018). The NCI Transcriptional Pharmacodynamics Workbench: A Tool to Examine Dynamic Expression Profiling of Therapeutic Response in the NCI-60 Cell Line Panel. *Cancer Research* 78**,** 6807-6817.

Papatheodorou, I., Moreno, P., Manning, J., Fuentes, A.M.-P., George, N., Fexova, S., Fonseca, N.A., Füllgrabe, A., Green, M., Huang, N., Huerta, L., Iqbal, H., Jianu, M., Mohammed, S., Zhao, L., Jarnuczak, A.F., Jupp, S., Marioni, J., Meyer, K., Petryszak, R., Prada medina, C.A., Talavera-López, C., Teichmann, S., Vizcaino, J.A., and Brazma, A. (2019). Expression Atlas update: from tissues to single cells. *Nucleic Acids Research*.

Pépin, G., De Nardo, D., Rootes, C.L., Ullah, T.R., Al-Asmari, S.S., Balka, K.R., Li, H.-M., Quinn, K.M., Moghaddas, F., Chappaz, S., Kile, B.T., Morand, E.F., Masters, S.L., Stewart, C.R., Williams, B.R.G., Gantier, M.P., and Horner, S.M. (2020). Connexin-Dependent Transfer of cGAMP to Phagocytes Modulates Antiviral Responses. *mBio* 11.

Pepin, G., Ferrand, J., Honing, K., Jayasekara, W.S., Cain, J.E., Behlke, M.A., Gough, D.J., Br, G.W., Hornung, V., and Gantier, M.P. (2016). Cre-dependent DNA recombination activates a STING-dependent innate immune response. *Nucleic Acids Res* 44**,** 5356-5364.

Ramanjulu, J.M., Pesiridis, G.S., Yang, J., Concha, N., Singhaus, R., Zhang, S.-Y., Tran, J.-L., Moore, P., Lehmann, S., Eberl, H.C., Muelbaier, M., Schneck, J.L., Clemens, J., Adam, M., Mehlmann, J., Romano, J., Morales, A., Kang, J., Leister, L., Graybill, T.L., Charnley, A.K., Ye, G.,

Nevins, N., Behnia, K., Wolf, A.I., Kasparcova, V., Nurse, K., Wang, L., Puhl, A.C., Li, Y., Klein, M., Hopson, C.B., Guss, J., Bantscheff, M., Bergamini, G., Reilly, M.A., Lian, Y., Duffy, K.J., Adams, J., Foley, K.P., Gough, P.J., Marquis, R.W., Smothers, J., Hoos, A., and Bertin, J. (2018). Design of amidobenzimidazole STING receptor agonists with systemic activity. *Nature* 564**,** 439-443.

Sarvestani, S.T., Tate, M.D., Moffat, J.M., Jacobi, A.M., Behlke, M.A., Miller, A.R., Beckham, S.A., Mccoy, C.E., Chen, W., Mintern, J.D., O'keeffe, M., John, M., Williams, B.R., and Gantier, M.P. (2014). Inosine-mediated modulation of RNA sensing by Toll-like receptor 7 (TLR7) and TLR8. *J Virol* 88**,** 799-810.
